# A Method of Making a Gold Inlay

**Published:** 1907-06-15

**Authors:** Henry Barnes

**Affiliations:** Cleveland, Ohio


### A Method of Making a Gold Inlay.

BY HENRY BARNES, M.D., CLEVELAND, OHIO.

It is sufficient to say, for cavity preparation, that there are two important mechanical principles to be observed: First, that the cavity preparation must provide a sufficent draft to provide for the easy withdrawal of the matrix from the cavity. Second, sufficient anchorage must be made to withstand the strain of mastication, and provide firm retention of the inlay within the cavity. When the first is not possible cement may be used to fill deep undercuts or pits caused by decay. The cavity being prepared, take a platinum foil one and one-half thousands gauge or fifteen ten-thousandths. This gives a matrix with much more stiffness and rigidity than can be made by the one-thousandths gauge. Cut piece large enough to cover bottom of cavity and extend well over margins. Now have suitably prepared sticks, pine or other wood six or eight inches in lenght, shaped at one end to fit the cavity, gently force platinum into the cavity with the stick, rubbing the same over all surfaces, allowing the foil to fold over margins if necessary. Burnish well into place, and over margins. Remove matrix and anneal, replacing same in cavity, and do as before. Do this until the matrix is fairly well in place. Now take uncured red label rubber, which is the rubber used to label the ordinary rubber garden hose, and cut piece large enough to fill cavity, and much more, then direct patient to close teeth upon same. forcing down upon it for a few minutes, and kneading the rubber between the teeth, care being taken to caution the patient never to remove the teeth from contact with the rubber, as the matrix may be withdrawn and spoilt; have patient carefully open the jaws, holding your finger upon the rubber, and remove rubber and matrix. The matrix may be broken in places, but if this is not over the margins no harm is done. Place matrix on charcoal block, and have pure gold plate cut into pieces, two or three of which place in matrix; if matrix is broken in body of cavity, melt one or two pieces of gold into a ball, and place same over break, sweating same into matrix, build up piece by piece, but do not at any time use large flame, as the secret of success is in sweating the gold into place, otherwise you will flow your gold over the outside.

The inlay unfinished may be tried in the cavity at any time to determine the exact amount of gold required. No investment is used; nothing to prevent the gold from flowing on reverse side of matrix or elsewhere; no borax is used, except at times, as in sweating you may not flow your gold sufficiently well over margins, in which case a tooth pick is dipped in vaseline borax, and a film is spread over margin desired to be covered; very small pieces of gold may be used for this purpose. If you have followed the technique you will have a gold inlay about the exact shape desired. The inlay being of pure gold, its margins may be burnished into place before and after setting, a very strong point in favor of pure gold.

